# ATF4 and SLC7A11 as biomarkers predicting progression and prognosis in lung adenocarcinoma

**DOI:** 10.3389/fonc.2026.1969897

**Published:** 2026-09-28

**Authors:** Yanjie Liu, Dongli Guo, Daojuan Li, Jing Jin

**Affiliations:** 1 Department of Thoracic Surgery, Fourth Hospital of Hebei Medical University, Shijiazhuang, China; 2 Cancer Institute, Fourth Hospital of Hebei Medical University, Shijiazhuang, China

**Keywords:** ATF4, lung adenocarcinoma, prognosis, prognostic prediction model, SLC7A11

## Abstract

**Background:**

Lung adenocarcinoma (LUAD) is the most prevalent subtype of non-small cell lung cancer, yet reliable biomarkers for stratifying early-stage patients at high risk of progression and poor prognosis remain limited.

**Methods:**

We enrolled 168 LUAD patients who underwent surgery at The Fourth Hospital of Hebei Medical University. ATF4 and SLC7A11 expressions were assessed by immunohistochemistry. Associations with clinicopathological features were evaluated by χ²/Fisher’s exact tests; univariate and multivariate Cox regression analyses of overall survival (OS) and metastasis-free survival (MFS) were performed, and model discrimination was quantified by the concordance index (C-index).

**Results:**

ATF4 was nuclear-positive in 55.4% of tumors, and SLC7A11 was cytoplasmic-positive in 59.5% of tumors. Neither ATF4 nor SLC7A11 was positively expressed in the stromal cells. The expression of ATF4 and SLC7A11 in tissues with pathological necrosis was enhanced. Both ATF4 and SLC7A11 correlated with aggressive features (more aggressive pathological subtypes with malignant potential, advanced clinical stage, lymph node metastasis, poor differentiation, pleural invasion, and pathological necrosis). ATF4 expression was also associated with lymphatic/vascular invasion. ATF4 (HR = 1.533, 95% CI, 1.040-2.259; *P* = 0.031) and SLC7A11 (HR = 1.527, 95% CI, 1.013-2.303; *P* = 0.043) were both independent prognostic factors for OS. As a combined indicator, ATF4 and SLC7A11 can enhance the C-index of the prognostic prediction model, and either positive for ATF4 or SLC7A11 have the highest C-index, regardless of whether it is for OS or MFS.

**Conclusion:**

ATF4/SLC7A11 are clinically useful prognostic biomarkers in LUAD. Either ATF4- or SLC7A11-positive expression appears to yield enhanced predictive value and may help identify high-risk patients for intensified surveillance.

## Introduction

Lung cancer remains the most commonly diagnosed malignancy and the leading cause of cancer-related mortality worldwide, imposing a severe burden on public health. According to the latest epidemiological data from the International Agency for Research on Cancer (IARC), lung cancer accounts for the highest number of new cancer cases and cancer deaths globally (1). Non-small cell lung cancer (NSCLC) constitutes approximately 85% of all lung cancer cases, among which lung adenocarcinoma (LUAD) is the predominant histological subtype, accounting for 40% to 50% of all lung cancers (2). Notably, the incidence of LUAD has risen continuously in recent years, particularly in female and nonsmoking populations. Despite substantial advances in surgical resection, targeted therapy, and immune checkpoint inhibitors, a considerable proportion of LUAD patients are diagnosed at advanced stages with lymph node invasion or distant metastasis, resulting in unsatisfactory long-term survival outcomes (3). The well-recognized independent prognostic factors for LUAD include TNM stage, pathological type, etc. In addition to traditional clinical indicators, emerging pathological microenvironmental characteristics have also been proven to affect tumor progression and clinical prognosis (4). Spontaneous intratumoral pathological necrosis, a common histological phenomenon induced by tumor hypoxia and nutrient deprivation, has been identified as a novel adverse prognostic biomarker in surgically resected LUAD (5). Intratumoral pathological necrosis is a type of passive, nonprogrammed cell death that is distinct from regulated necroptosis. Necrotic cells release abundant damage-associated molecular patterns, which remodel the tumor microenvironment into a proinflammatory and immunosuppressive state, facilitating angiogenesis, extracellular matrix remodeling, and tumor immune evasion, which ultimately contributes to LUAD malignant progression and distant metastasis (6).

Activating transcription factor 4 (ATF4) is a core downstream effector of the integrated stress response. Tumor cells within the necrotic core undergo irreversible passive death, whereas viable cancer cells residing in the perinecrotic border are persistently challenged by endoplasmic reticulum stress, ROS accumulation, and iron overload, triggering the activation of ATF4, the master effector of the integrated stress response (7). Upon stress stimulation, ATF4 translocates into the nucleus and transcriptionally activates SLC7A11 via binding to the amino acid response element on its promoter (8). SLC7A11 is essential for glutathione (GSH) biosynthesis. Increased GSH content enhances the intracellular antioxidant capacity, eliminates lipid reactive oxygen species, and effectively inhibits ferroptosis, an iron-dependent form of programmed necrotic cell death (9). Nevertheless, the spatial correlation between ATF4/SLC7A11 expression and intratumoral pathological necrosis, as well as the clinical prognostic significance of the ATF4-SLC7A11 regulatory axis in LUAD, remains insufficiently clarified in clinical cohorts.

In this study, we detected the expression and subcellular localization of ATF4 and SLC7A11 in surgically resected treatment-naive primary LUAD tissues. We further analyzed the correlations of ATF4 and SLC7A11 expression with intratumoral necrosis, clinicopathological characteristics, and patient prognosis. This study aimed to explore the clinical evidence and potential molecular mechanism through which the ATF4/SLC7A11 axis mediates necrotic microenvironment-driven LUAD progression, providing novel biomarkers and therapeutic targets for LUAD prognostic evaluation and clinical intervention.

## Materials and methods

### Patients

### Ethics statement

This study was approved by the Institutional Human Ethics Committee (2026KS120) of The Fourth Hospital of Hebei Medical University (also referred to as Hebei Cancer Hospital). Prior written informed consent was obtained from all patients. All samples were anonymously coded in accordance with local ethical guidelines.

### Data collection

300 lung cancer patients were recruited through a consecutive enrollment process, and 168 of them with lung adenocarcinoma were selected for this analysis. All patients underwent surgical treatment at The Fourth Hospital of Hebei Medical University (also referred to as Hebei Cancer Hospital). The specimens collected included lung cancer tissues and adjacent non-tumor tissues. All the patients were diagnosed with lung adenocarcinoma through pathological examination. Personal data of the patients, including sex, age, and tumor-related data, were extracted from the medical records. Detailed patient information is provided in **Table 1**.

**Table 1:** Patient and tumor characteristics (n=168).

Characteristic	Cases (n=168)
Sex	
Male	78
Female	90
Age (Male, Female)	
≥60	92 (46, 46)
<60	76 (32, 44)
BI (Male, Female)	
0	99 (45, 54)
0–1000	41 (16, 25)
1000+	28 (17, 11)
Location (Male, Female)	
central type	11 (6, 5)
peripheral type	157 (72, 85)
Histopathological subtype (Male, Female)	
MIA/LPA	17 (6, 11)
APA/PPA	113 (57, 56)
SPA/MPA	38 (15, 23)
Clinical stage (Male, Female)	
I	16 (7, 9)
II	73 (37, 36)
III	79 (34, 45)
N (Male, Female)	
Positive	80 (34, 46)
Negative	88 (44, 44)
Differentiation (Male, Female)	
Well	38 (16, 22)
Moderately	91 (44, 47)
Poorly	39 (18, 21)
Pleural invasion (Male, Female)	
Yes	78 (38, 40)
No	90 (40, 50)
Lymphatic/Vascular invasion (Male, Female)	
Yes	26 (17, 9)
No	142 (61, 81)
Pathological necrosis (Male, Female)	
Yes	49 (21, 28)
No	119 (57, 62)

Follow-up evaluations were performed according to the standard follow-up system of the hospital every 6 months after patients were discharged from the hospital. The deadline for follow-up evaluations was June 1, 2026. All patients were followed from the date of histological diagnosis until death or the last day of follow-up and were followed for at least 12 months. The overall survival (OS) period was calculated from the date of surgery until the date of death or the end of follow-up. Metastasis-free survival (MFS) is defined as time from surgery to cancer recurrence or metastasis, death, or the end of the follow-up. The patients in this study were all lung adenocarcinoma patients, with a median follow-up period of 35 months and 142 outcome events for OS, whereas the median follow-up period was 32 months, with 142 outcome events for MFS.

Inclusion criteria: Patients pathologically diagnosed with primary lung adenocarcinoma. Lung adenocarcinoma was classified according to the 8th IASLC TNM staging system. Exclusion criteria: Patients receiving neoadjuvant chemotherapy, radiotherapy, targeted therapy, or immunotherapy before surgery. Patients lost to follow-up with incomplete survival information. Metastatic adenocarcinoma to the lung (non-primary lung cancer).

### Immunohistochemistry

Paraffin sections (4 µm) were deparaffinized and rehydrated, followed by antigen retrieval using 0.02 M EDTA buffer. The sections were treated with 3% H_2_O_2_, blocked with 5% normal goat serum, and then incubated with a monoclonal anti-ATF4 (1:500; Abcam, ab216839) or anti-SLC7A11 (1:1000; Abcam, ab307601) antibody overnight at 4 °C. The antibody was diluted in phosphate-buffered saline (PBS) buffer containing 5% normal goat serum. For negative controls, adjacent sections were incubated with 5% normal goat serum in the absence of the primary antibody. The sections were then incubated with horseradish peroxidase-conjugated anti-rabbit IgG for 45 minutes at 37 °C and visualized using diaminobenzidine tetrahydrochloride. Stained slides were examined under a light microscope and evaluated by two pathologists who were blinded to the clinical data.

Samples were scored according to the percentage and intensity scores of positively stained tumor cells. Percentage score was as follows: 1 point if the proportion of positive cells was less than 25%; 2 points if it was between 25% and 50%; 3 points if it was between 50% and 75%; and 4 points if it was between 75% and 100%. The staining intensity is based on the depth of staining as the standard. Unstained samples are scored as 0 points, lightly stained samples are scored as 1 point, moderately stained samples are scored as 2 points, and heavily stained samples are scored as 3 points. Finally, the product of these two scores was calculated as the immunohistochemistry (IHC) score of the protein. If the score was greater than or equal to 2, it indicated positive protein expression.

### Statistical analysis

Statistical analyses were performed using R software (version 4.5.1, R Foundation for Statistical Computing, Vienna, Austria). The R packages including survival (v3.8-3), glmnet (v5.0), rms (v8.1-1), timeROC (v0.4-11) were applied for Cox proportional-hazards regression, LASSO regression, and nomogram construction, and time-dependent ROC analysis, respectively. Multicollinearity among candidate predictors was assessed by calculating the variance inflation factor (VIF). Comparisons of categorical variables were performed using the χ² test; Fisher’s exact test was adopted if any expected cell frequency was less than 5. Continuous variables were assessed with independent samples t-test or Mann–Whitney U test based on the normality of data distribution. For quantitative data, the independent-samples t-test was applied for normally distributed variables (between-group homogeneity of variance test, α=0.05; Satterthwaite-corrected t-test for heterogeneous variance), whereas the Mann-Whitney U test was used for non-normally distributed data and ordinal categorical variables. Variable Selection and Model Construction: Variables with P<0.10 in univariate Cox regression were screened, and candidate predictors for multivariate Cox regression were selected based on prior literature and clinical experience. The forward likelihood ratio (Forward LR) method was used for further variable selection to construct the prognostic model. Time-dependent receiver-operating characteristic (ROC) curves and corresponding AUC values for survival endpoints (OS and MFS) were generated via the timeROC package. The cumulative-incidence approach was used to handle right-censored survival data; patients censored before the target time point were excluded from the risk-set for the corresponding time-specific AUC calculation. Time-specific AUCs were calculated at the median follow-up time of 35 months for OS and 32 months for MFS. The C-index quantified the overall discriminative ability of prognostic models across the entire follow-up period, whereas time-dependent AUC evaluated predictive performance at a predefined single time point. GraphPad Prism (version 10.2.0, GraphPad Software, USA) was used for plotting and basic statistical tests including Student’s t-test and one-way ANOVA. *P* < 0.05 was considered statistically significant.

## Results

### Clinical data

A total of 168 lung adenocarcinoma patients were enrolled in this study, and the cohort consisted of 78 males and 90 females, with a median age of 60.5 years (range: 40–75 years). A total of 54.8% of the patients were older than 60 years, 47.6% had lymph node metastasis (N), 46.4% had pleural invasion, 15.5% had lymphatic/vascular invasion, and 29.2% had pathological necrosis. More than half of the tumors (54.2%) were moderately differentiated, followed by poorly differentiated (23.2%) and well-differentiated (22.6%). Most lung adenocarcinomas are peripheral (93.5%) lesions located in the lung periphery distal to segmental bronchi. There were 16 patients (9.5%) with c-stage I disease, 73 patients (43.5%) with c-stage II disease, and 79 patients (47.0%) with c-stage III disease. The predominant histopathological subtype was invasive adenocarcinoma acinar predominant (APA, 50.6%), followed by invasive adenocarcinoma solid predominant (SPA, 17.9%) and invasive adenocarcinoma papillary predominant (PPA, 16.7%). The number of other histopathological subtypes was very small (invasive adenocarcinoma lepidic predominant, LPA, 10 patients; minimally invasive adenocarcinoma, MIA, 7 patients; invasive adenocarcinoma micropapillary predominant, MPA, 8 patients), and we combined them based on the malignancy degree of the pathological types (**Table 1**). Additionally, among these patients, 69 were smokers. We grouped the patients based on the Brinkman index (BI) (**Table 1**).

### Expression of ATF4 and SLC7A11 in lung adenocarcinoma tissues

Immunohistochemical staining of lung adenocarcinoma tissues revealed that ATF4 was mainly localized in the nucleus rather than in the cytoplasm of lung adenocarcinoma cells (**Figures 1A–C**), with a positive rate of 55.4%. SLC7A11 was primarily localized to the cytoplasm without obvious nuclear staining (**Figure 1A–C**), with a positive expression rate of 59.5%. Neither ATF4 nor SLC7A11 was positively expressed in the stromal cells (**Figure 1A–C**). The expression levels of ATF4 and SLC7A11 are consistent. Usually, in tissues that are positive for ATF4, SLC7A11 is also expressed positively (**Table 2**). We also examined the expression levels of ATF4 and SLC7A11 in tissues with pathological necrosis. The results showed that among the 49 lung adenocarcinoma tissues with pathological necrosis, 39 cases showed positive expression of ATF4, and the positive rate (79.6%) was much higher than that of the lung adenocarcinoma tissues without pathological necrosis (45.4%) (**Table 2**). SLC7A11 also showed the same trend. Among lung adenocarcinoma tissues with pathological necrosis, 81.6% showed positive expression of SLC7A11, whereas among lung adenocarcinoma tissues without pathological necrosis, 50.4% had positive SLC7A11 expression (**Table 3**; **Figure 1C**).

**Figure 1:**
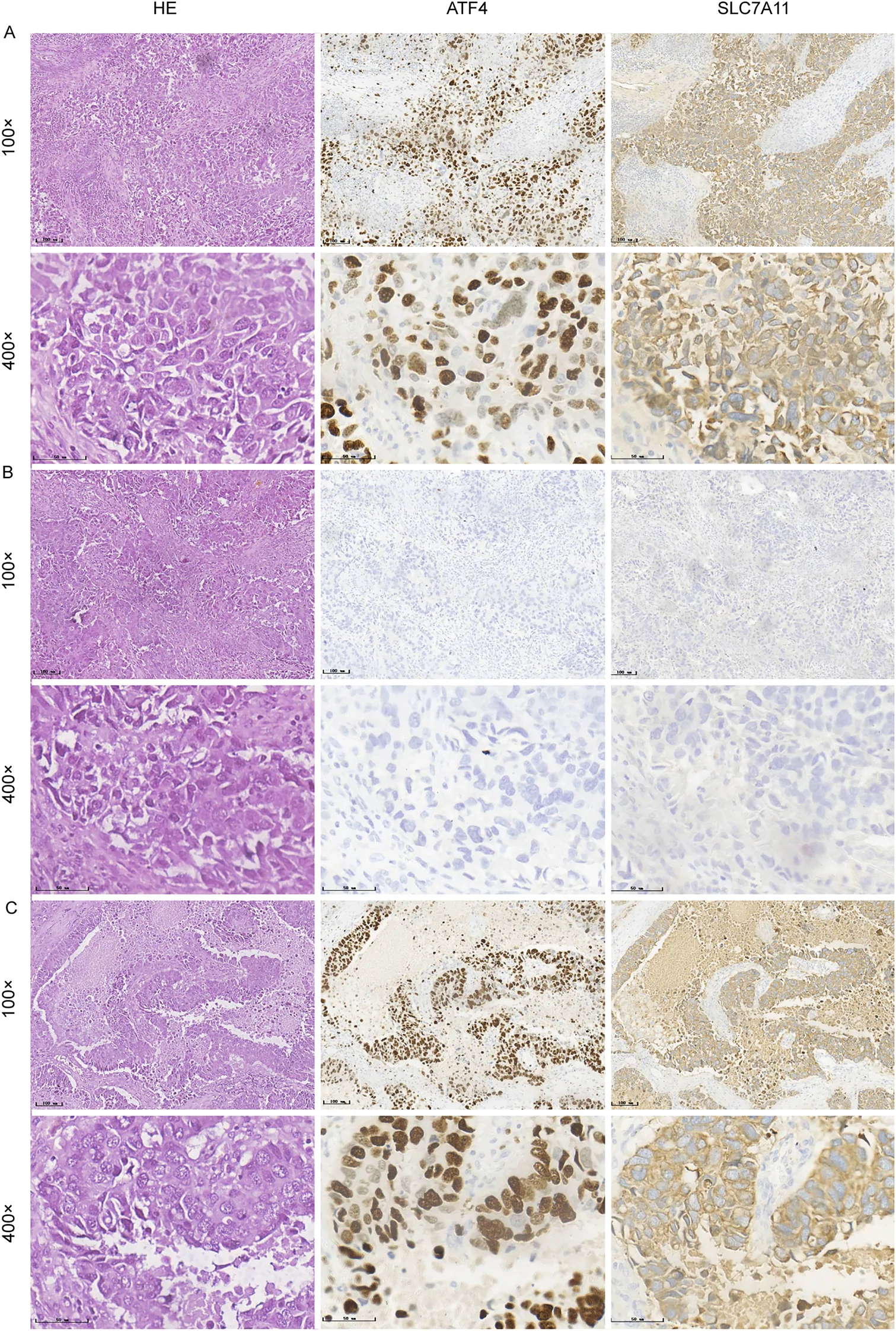
Representative immunohistochemical staining images of ATF4 and SLC7A11 in lung adenocarcinoma tissues. **(A)** ATF4 and SLC7A11 are positive in some tissues and are located in the nucleus and cytoplasm respectively; **(B)** ATF4 and SLC7A11 are negative in some tissues; **(C)** ATF4 and SLC7A11 are positive in lung adenocarcinoma tissues with pathological necrosis.

**Table 2:** Associations between ATF4 expression and patient characteristics.

Characteristic	ATF4+ No.	ATF4+ %	ATF4− No.	ATF4− %	χ²	*P*
Sex					0.321	0.571
Male	45	57.7	33	42.3		
Female	48	53.3	42	46.7		
Age					0.917	0.338
≥60	54	58.7	38	41.3		
<60	39	51.3	37	48.7		
BI					4.247	0.120
0	59	59.6	40	40.4		
0–1000	17	41.5	24	58.5		
1000+	17	60.7	11	39.3		
Location						0.966*
central type	6	54.5	5	45.5		
peripheral type	87	55.4	70	44.6		
Histopathological subtype					23.600	<0.001
MIA/LPA	1	5.9	16	94.1		
APA/PPA	63	55.8	50	44.2		
SPA/MPA	29	76.3	9	23.7		
Clinical stage					27.753	<0.001
I	2	12.5	14	87.5		
II	32	43.8	41	56.2		
III	59	74.7	20	25.3		
**N**					30.301	<0.001
Positive	62	77.5	18	22.5		
Negative	31	35.2	57	64.8		
Differentiation					11.308	0.004
Well	12	31.6	26	68.4		
Moderately	56	61.5	35	38.5		
Poorly	25	64.1	14	35.9		
Pleural invasion					4.506	0.034
Yes	50	64.1	28	35.9		
No	43	47.8	47	52.2		
Lymphatic/Vascular invasion					8.038	0.005
Yes	21	80.8	5	19.2		
No	72	50.7	70	49.3		
Pathological necrosis					16.440	<0.001
Yes	39	79.6	10	20.4		
No	54	45.4	65	54.6		
SLC7A11					7.468	0.006
Positive	64	64.0	36	36.0		
Negative	29	42.6	39	57.4		

*Fisher’s exact test.

**Table 3:** Associations between SLC7A11 expression and patient characteristics.

Characteristic	SLC7A11+ No.	SLC7A11+ %	SLC7A11− No.	SLC7A11− %	χ²	*P*
Sex					0.018	0.893
Male	46	59.0	32	41.0		
Female	54	54.0	36	36.0		
Age					0.500	0.480
≥60	57	62.0	35	38.0		
<60	43	56.6	33	43.4		
BI					1.617	0.446
0	61	61.6	38	38.4		
0–1000	21	51.2	20	48.8		
1000+	18	64.3	10	35.7		
Location					2.621	0.105
central type	4	36.4	7	63.6		
peripheral type	96	61.1	61	38.9		
Histopathological subtype						<0.001*
MIA/LPA	0	0.0	17	100.0		
APA/PPA	65	57.5	48	42.5		
SPA/MPA	35	92.1	3	7.9		
Clinical stage					34.906	<0.001
I	2	12.5	14	87.5		
II	34	46.6	39	53.4		
III	64	81.0	15	19.0		
N					33.464	<0.001
Positive	66	82.5	14	17.5		
Negative	34	38.6	54	61.4		
Differentiation					15.373	<0.001
Well	13	34.2	25	65.8		
Moderately	57	62.6	34	37.4		
Poorly	30	76.9	9	23.1		
Pleural invasion					11.101	0.001
Yes	57	73.1	21	26.9		
No	43	47.8	47	52.2		
Lymphatic/Vascular invasion					1.203	0.273
Yes	18	69.2	8	30.8		
No	82	57.7	60	42.3		
Pathological necrosis					14.035	0.001
Yes	40	81.6	9	18.4		
No	60	50.4	59	49.6		

* Fisher’s exact test.

Expression of ATF4 and SLC7A11 and Their Correlations with Clinicopathological Characteristics

We collected clinical specimens from patients with lung adenocarcinoma and analyzed them. The chi-square (χ²) test was used to evaluate the associations of ATF4 and SLC7A11 expression with clinicopathological parameters. The results demonstrated that ATF4 expression was significantly correlated with histopathological subtype, clinical stage, lymph node metastasis, differentiation, pleural invasion, lymphatic/vascular invasion and pathological necrosis. Patients positive for ATF4 exhibited more aggressive pathological subtypes with malignant potential, more advanced clinical stage, lymph node metastasis, poorer differentiation, pleural invasion, lymphatic/vascular invasion and pathological necrosis (**Table 2**). SLC7A11 expression was associated with histopathological subtype, clinical stage, lymph node metastasis, differentiation, pleural invasion and pathological necrosis. Patients who were positive for SLC7A11 showed more aggressive pathological subtypes with malignant potential, more advanced clinical stage, lymph node metastasis, poorer differentiation, pleural invasion and pathological necrosis (**Table 3**).

Correlation Between ATF4 and SLC7A11 Expression Levels and Patient Prognosis

We analyzed the associations of these factors with patient prognosis. Univariate analysis revealed that OS was associated with histopathological subtype, clinical stage, N, differentiation, pleural invasion, lymphatic/vascular invasion, pathological necrosis, and ATF4 and SLC7A11 expression, but not with sex, age, BI, or tumor location (**Table 4**; **Figure 2A**). MFS was associated with histopathological subtype, clinical stage, N, differentiation, pleural invasion, lymphatic/vascular invasion, pathological necrosis, and ATF4 and SLC7A11 expression, but not with sex, age, BI, or tumor location (**Table 5**; **Figure 2B**). We also evaluated the correlation between the combined expression of ATF4 and SLC7A11 and patient prognosis. Combined positivity for both ATF4- and SLC7A11-positive tumors, as well as either ATF4- or SLC7A11-positive tumors, was significantly associated with reduced OS and MFS (**Figures 2A, B**).

**Table 4:** Univariate cox regression analysis between these factors and the OS of patients.

Factor	B	HR (95% CI)	*P*
Sex	−0.118	0.889 (0.613–1.289)	0.534
Age	0.253	1.288 (0.921–1.799)	0.139
BI	0.185	1.204 (0.966–1.500)	0.099
Location	0.777	2.175 (0.933–5.067)	0.072
Histopathological subtype	2.554	12.853 (4.050–40.796)	<0.001
Clinical stage	1.563	4.773 (3.458–6.588)	<0.001
N	2.121	8.336 (5.524–12.580)	<0.001
Differentiation	0.593	1.810 (1.428–2.295)	<0.001
Pleural invasion	1.165	3.205 (2.250–4.565)	<0.001
Lymphatic/Vascular invasion	0.502	1.653 (1.067–2.560)	0.024
Pathological necrosis	1.661	5.267 (3.488–7.954)	<0.001
ATF4	0.938	2.555 (1.808–3.612)	<0.001
SLC7A11	1.217	3.904 (2.463–6.190)	<0.001

**Figure 2:**
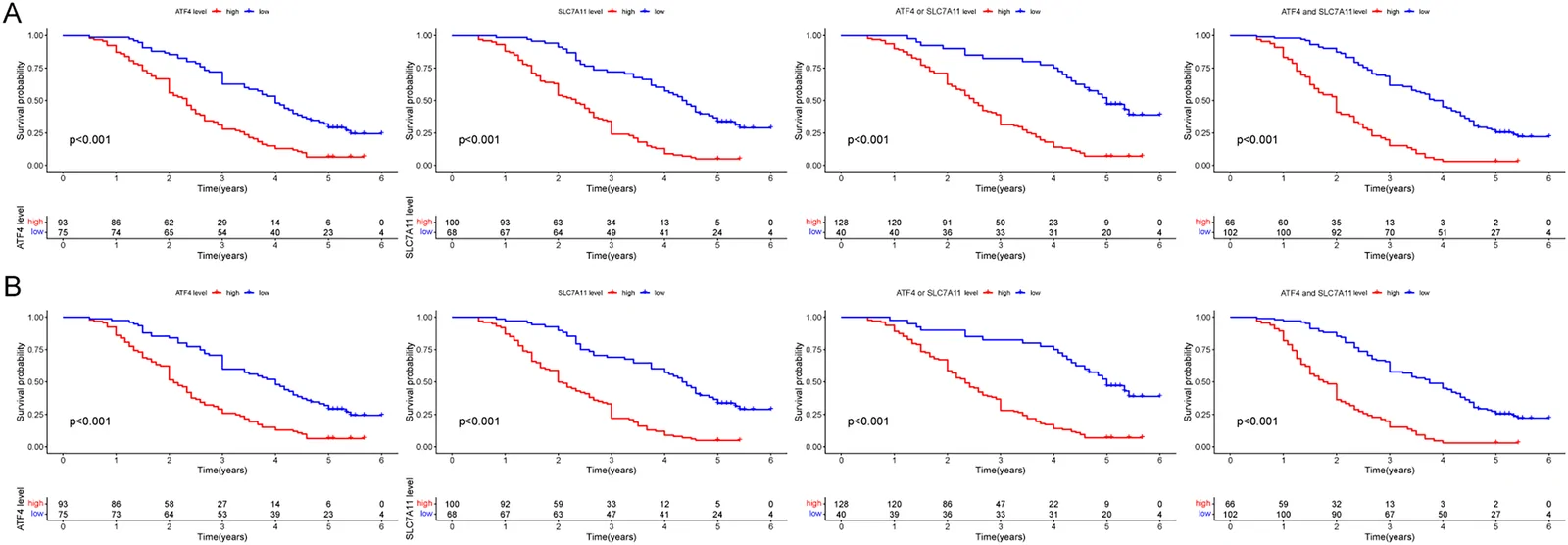
Clinical significance of ATF4 and SLC7A11 in the OS and MFS of patients with lung adenocarcinoma. **(A)** The expression levels of ATF4 and SLC7A11 and their relationship as a combined indicator with OS; **(B)** The expression levels of ATF4 and SLC7A11 and their relationship as a combined indicator with MFS.

**Table 5:** Univariate cox regression analysis between these factors and the MFS of patients.

Factor	B	HR (95% CI)	*P*
Sex	−0.151	0.860 (0.619–1.196)	0.371
Age	0.254	1.290 (0.923–1.802)	0.136
BI	0.199	1.220 (0.979–1.520)	0.077
Location	0.724	2.062 (0.909–4.678)	0.083
Histopathological subtype	1.107	3.026 (2.238–4.093)	<0.001
Clinical stage	1.548	4.704 (3.403–6.502)	<0.001
N	2.021	7.549 (5.002–11.392)	<0.001
Differentiation	0.574	1.775 (1.400–2.251)	<0.001
Pleural invasion	1.132	3.103 (2.181–4.416)	<0.001
Lymphatic/Vascular invasion	0.499	1.647 (1.065–2.548)	0.025
Pathological necrosis	1.673	5.326 (3.529–8.038)	<0.001
ATF4	0.918	2.504 (1.772–3.540)	<0.001
SLC7A11	1.179	3.250 (2.251–4.692)	<0.001

The non-biomarker variables with P values less than 0.10 in univariate analysis were further entered into multivariate analysis, which identified the BI, clinical stage, pleural invasion, differentiation, and pathological necrosis as independent prognostic risk factors for OS and the BI, clinical stage, pleural invasion, differentiation, and pathological necrosis as independent prognostic risk factors for MFS (**Table 6**). If ATF4 and SLC7A11 were included in the multivariate analysis, the results showed that the independent risk factors for OS were BI, clinical stage, pleural invasion, differentiation, pathological necrosis, and ATF4 and SLC7A11, and the independent risk factors for MFS were BI, clinical stage, pleural invasion, and pathological necrosis (**Table 7**). We calculated the VIF to assess multicollinearity among variables. All VIF values were below 5, with detailed results presented in **Supplementary Table S1**. If ATF4 and SLC7A11 were used as combined indicators for multivariate analysis, both the “both ATF4- and SLC7A11-positive” group (HR = 1.541; 95% CI, 1.012–2.348; *P* = 0.044) and the “either ATF4- or SLC7A11-positive” group (HR = 2.022; 95% CI, 1.222–3.344; *P* = 0.006) can be included in the OS model, whereas the MFS group can only be included in the “either ATF4- or SLC7A11-positive” group (HR = 2.019; 95% CI, 1.204–3.386; *P* = 0.008).

**Table 6:** Multivariate cox regression analysis between non-biomarker factors and OS and MFS of patients (non-biomarker model).

Outcome	Factor	B	HR (95% CI)	*P*
OS				
	Clinical stage	1.640	5.155 (3.607–7.368)	<0.001
	Pleural invasion	0.747	2.111 (1.441–3.092)	<0.001
	Differentiation	0.342	1.408 (1.095–1.811)	0.008
	Pathological necrosis	1.426	4.163 (2.703–6.413)	<0.001
	BI	0.261	1.298 (1.046–1.611)	0.018
MFS				
	Clinical stage	1.742	5.709 (3.958–8.235)	<0.001
	Pleural invasion	0.797	2.219 (1.517–3.246)	<0.001
	Differentiation	0.336	1.399 (1.092–1.792)	0.008
	Pathological necrosis	1.572	4.817 (3.125–7.427)	<0.001
	BI	0.341	1.407 (1.130–1.752)	0.002

**Table 7:** Multivariate cox regression analysis between all factors and the OS and MFS of patients (full model including ATF4 and SLC7A11).

Outcome	Factor	B	HR (95% CI)	*P*
OS				
	BI	0.313	1.368 (1.096–1.706)	0.006
	Clinical stage	1.514	4.547 (3.127–6.613)	<0.001
	Pleural invasion	0.702	2.018 (1.371–2.970)	<0.001
	Differentiation	0.260	1.297 (1.000–1.683)	0.050
	Pathological necrosis	1.289	3.630 (2.337–5.638)	<0.001
	ATF4	0.427	1.533 (1.040–2.259)	0.031
	SLC7A11	0.423	1.527 (1.013–2.303)	0.043
MFS				
	BI	0.381	1.463 (1.169–1.831)	0.001
	Clinical stage	1.631	5.107 (3.485–7.483)	<0.001
	Pleural invasion	0.746	2.108 (1.435–3.097)	<0.001
	Pathological necrosis	1.452	4.271 (2.747–6.641)	<0.001

We subsequently constructed a prognostic prediction model for OS, and the C-index of the prognostic model solely based on clinical factors was 0.835 (95% CI: 0.808–0.862). Incorporating ATF4 and SLC7A11 into the prognostic model increased the C-index to 0.839 (95% CI: 0.812–0.868). When markers were combined, the C-index was 0.838 (95% CI: 0.812–0.865) for both the ATF4- and SLC7A11-positive samples and was 0.840 (95% CI: 0.811–0.869) for either ATF4- or SLC7A11-positive samples (**Figure 3A**). For MFS, the C-index of the prognostic model based solely on clinical factors was 0.841 (95% CI: 0.816–0.867). Incorporating ATF4 and SLC7A11 into the prognostic model decreased the C-index to 0.832 (95% CI: 0.806–0.858). However, when the markers were combined, the C-index was 0.844 (95% CI: 0.819–0.870) for both the ATF4- and SLC7A11-positive samples and was 0.847 (95% CI: 0.821–0.875) for either ATF4- or SLC7A11-positive samples (**Figure 3B**). For both OS and MFS, the group defined as either ATF4- or SLC7A11-positive exhibited the optimal predictive performance when ATF4 and SLC7A11 were used as combined biomarkers.

**Figure 3:**
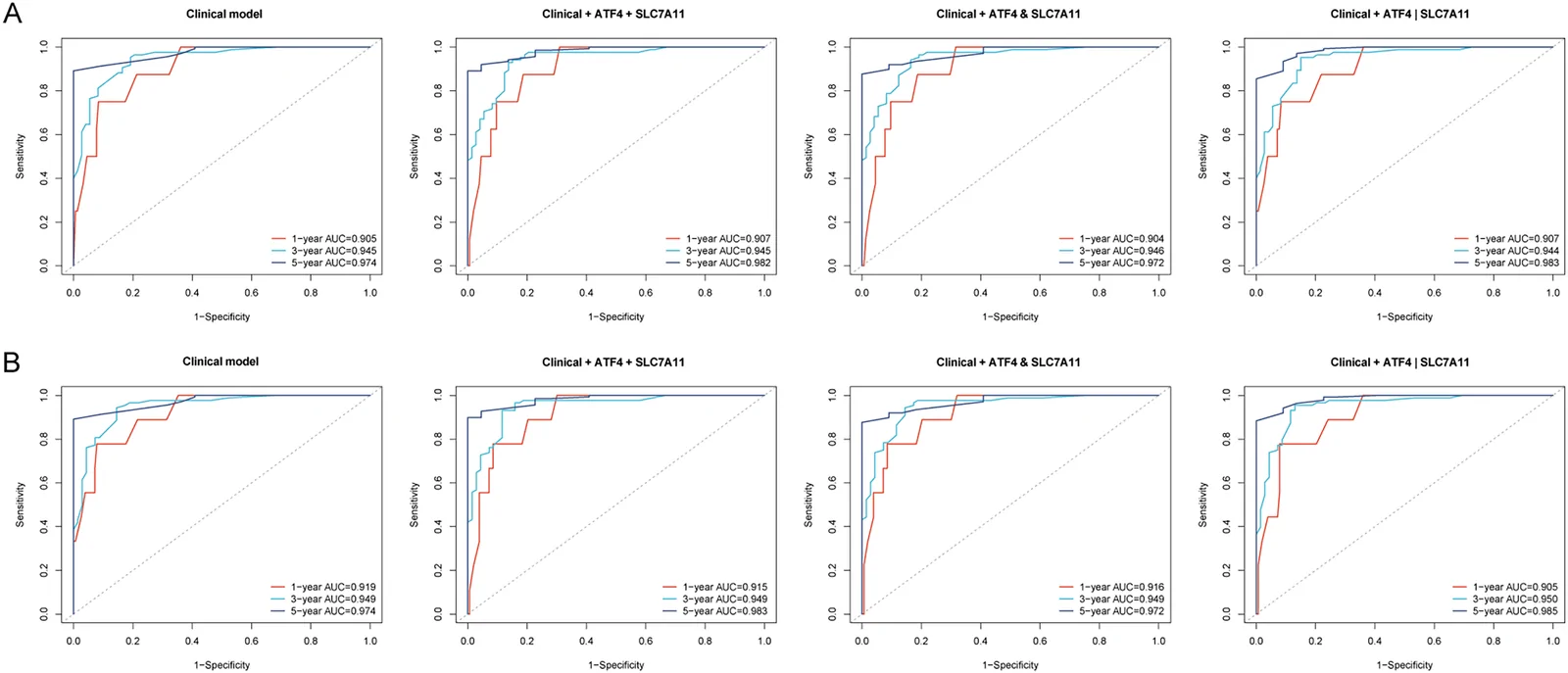
Prognostic value of ATF4 and SLC7A11 in predicting the prognosis of lung adenocarcinoma patients. **(A)** The expression levels of ATF4 and SLC7A11 and their predictive value as a combined indicator for OS; **(B)** The expression levels of ATF4 and SLC7A11 and their predictive value as a combined indicator for MFS. ATF4 and SLC7A11 (both ATF4 and SLC7A11 positive). ATF4 | SLC7A11 (either ATF4 or SLC7A11 positive).

## Discussion

In the present study, we retrospectively enrolled 168 patients with surgically resected lung adenocarcinoma to investigate the expression pattern, clinicopathological correlation and prognostic implication of ATF4 and SLC7A11. Immunohistochemical results demonstrated that ATF4 was predominantly localized in the nucleus, whereas SLC7A11 was mainly localized to the cytoplasm of tumor cells, with no positive staining observed in stromal components. The expression status of ATF4 and SLC7A11 exhibited obvious consistency, and tumors with pathological necrosis displayed markedly higher positive rates of both proteins. Mechanistically, ATF4 acts as a core transcription factor responding to endoplasmic reticulum stress and oxidative stress, and can transcriptionally activate SLC7A11 to enhance cystine uptake, maintain glutathione synthesis, and suppress ferroptosis in malignant cells (10, 11). Consistent with our findings, these studies have confirmed the phenomenon of coordinated expression of ATF4 and SLC7A11 in tumor tissues. Studies have shown that severe hypoxia, oxidative stress and metabolic disorder commonly exist in necrotic tumor regions, which may robustly trigger ATF4 upregulation and subsequently induce SLC7A11 expression to strengthen redox defense for surviving tumor cells within the adverse microenvironment (12, 13). This might explain part of the reason why the expression of ATF4 and SLC7A11 is elevated and is associated with pathological necrosis.

Our results revealed that positive ATF4 and SLC7A11 expression was significantly correlated with aggressive clinicopathological phenotypes, including advanced clinical stage, lymph node metastasis, poor differentiation, pleural invasion, and pathological necrosis. Tumors with high ATF4/SLC7A11 expression tended to be associated with more malignant histologic subtypes, such as solid predominant invasive adenocarcinoma. These clinical correlations support the oncogenic function of ATF4 and SLC7A11 in facilitating lung adenocarcinoma progression and invasive capacity (14, 15). Therefore, the detection of ATF4 and SLC7A11 expression may help identify patients with highly aggressive tumor phenotypes at initial diagnosis. Survival analyses further validated the prognostic value of the two molecules. Univariate Cox regression indicated that positive ATF4 and SLC7A11 expression were adverse prognostic factors for both OS and MFS. After incorporating biomarkers into multivariate models, single-ATF4 or SLC7A11 positivity remained an independent risk predictor for OS.

Given that single molecular markers often have limited predictive stability, we constructed two combined biomarker definitions: both positive for both ATF4 and SLC7A11 and positive for either ATF4 or SLC7A11. Multivariate results demonstrated that the either ATF4- or SLC7A11-positive group achieved consistent statistical significance for both OS and MFS endpoints, whereas dual positivity only maintained prognostic relevance for OS. Using “either marker positive” as the combined cutoff can identify more patients with activated ATF4/SLC7A11 signaling, avoiding the underestimation of high-risk populations caused by strict dual-positive criteria, which is consistent with the findings of previous studies showing that compared with single indicators, combined biomarker panels improve risk stratification (16).

We further constructed prognostic models and compared the discriminative capacity of different grouping strategies via the C-index. Adding ATF4 and SLC7A11 to conventional clinical models moderately improved predictive performance for OS. For MFS, when ATF4 and SLC7A11 were included in the model, the C-index decreased. This was because in the multivariate analysis, ATF4 and SLC7A11 were not independent risk factors for MFS, and their predictive effect might have been overshadowed by other more important factors such as clinical stage or pleural invasion. Importantly, for both OS and MFS, the prognostic model based on the either ATF4- or SLC7A11-positive group achieved the optimal C-index, outperforming models using single biomarkers or dual-positive criteria. These findings indicate that the “either-or” combined definition appears to be a favorable grouping strategy when ATF4 and SLC7A11 are utilized as joint prognostic biomarkers. From a biological perspective, ATF4 and SLC7A11 form a regulatory cascade rather than being fully dependent parallel markers; activation of any component of this axis is sufficient to initiate cytoprotective metabolic programs, which explains why any single positive status effectively reflects poor prognosis (17). Clinically, this simple IHC-based grouping strategy can be conveniently implemented in routine pathological reports to refine postoperative risk stratification beyond traditional clinicopathological parameters.

Several limitations of this study should be acknowledged. This was a single-center retrospective cohort and cohort includes only patients with stage I-III disease who underwent curative-intent surgical resection, the conclusions apply specifically to the surgically resectable early-to-middle-stage LUAD population. The sampling method used in this study was consecutive enrollment, and its representativeness might not be as good as that of random sampling. In addition, only internal comparisons of model performance were conducted; external independent cohort validation is required to confirm the generalizability of our combined biomarker model and to enhance the evidence by adding net reclassification improvement or integrated discrimination improvement data. Further prospective studies combining functional experiments are warranted to clarify how pathological necrosis regulates the ATF4/SLC7A11 pathway and to explore therapeutic strategies targeting this axis in high-risk lung adenocarcinoma patients defined by our grouping standard.

## Conclusion

ATF4 is expressed in the nuclei of lung adenocarcinoma cells and is associated with invasive characteristics (more aggressive pathological subtypes with malignant potential, advanced clinical stage, lymph node metastasis, poorer differentiation, pleural invasion, lymphatic/vascular invasion and pathological necrosis), and is an independent prognostic factor for OS. SLC7A11 is expressed in the cytoplasm of lung adenocarcinoma cells and is also related to invasive characteristics (more aggressive pathological subtypes with malignant potential, advanced clinical stage, lymph node metastasis, poorer differentiation, pleural invasion, and pathological necrosis), and is an independent prognostic factor for OS. As a combined indicator, ATF4 and SLC7A11 can enhance the C-index of the prognostic prediction model, and patients who are either positive for ATF4 or SLC7A11 have the highest C-index, regardless of whether it is for OS or MFS.

## Data Availability

The original contributions presented in the study are included in the article/**Supplementary Material**. Further inquiries can be directed to the corresponding author/s.
