# Supplementary material for: ATF4 and SLC7A11 as biomarkers predicting progression and prognosis in lung adenocarcinoma
Source: Front Oncol. 2026 Sep 28;16:1969897. doi: 10.3389/fonc.2026.1969897 (PMC13655137; doi:10.3389/fonc.2026.1969897)
Supplement: Supplementary file 1 [file Table1.docx]

Supplementary Table S1. VIF of candidate predictors for OS‑ and MFS‑based multivariate Cox regression models

| Model | Clinical stage | Pleural invasion | Differentiation grade | Necrosis | BI | ATF4 | SLC7A11 | ATF4 or SLC7A11 | ATF4 and SLC7A11 |
| --- | --- | --- | --- | --- | --- | --- | --- | --- | --- |
| OS | 1.129 | 1.11 | 1.065 | 1.148 | 1.149 | 1.128 | 1.101 | — | — |
|  | 1.075 | 1.101 | 1.033 | 1.116 | 1.063 | — | — | 1.023 | — |
|  | 1.116 | 1.094 | 1.097 | 1.158 | 1.108 | — | — | — | 1.23 |
| MFS | 1.144 | 1.059 | 1.079 | 1.125 | 1.137 | 1.133 | 1.084 | — | — |
|  | 1.091 | 1.054 | 1.034 | 1.092 | 1.058 | — | — | 1.03 | — |
|  | 1.136 | 1.052 | 1.095 | 1.119 | 1.095 | — | — | — | 1.177 |
